# The impact of the COVID-19 pandemic and pandemic-related policies on new firm creation: an analysis of the Italian case

**DOI:** 10.1007/s11187-022-00621-w

**Published:** 2022-04-20

**Authors:** Evila Piva, Massimiliano Guerini

**Affiliations:** Department of Management, Economics and Industrial Engineering, Politecnico Di Milano, via Lambruschini 4/b, 20156 Milano, Italy

**Keywords:** COVID-19, Disasters, New firm creation, Policy measures, H84, L26

## Abstract

This work contributes to disaster research by exploring the impact on new firm creation of the COVID-19 pandemic and the pandemic-related policies. We develop hypotheses on the individual and combined effects of pandemic severity and public policies aimed at controlling the spread of the disease (shutdown policies) or protecting the economy from its negative consequences (demand stimulus and firm support policies). Then, we test these hypotheses using data on Italy in the first and second 2020 pandemic waves. Results show that pandemic severity negatively affected new firm creation during the first wave. Shutdown policies had negative effects too, especially in the regions where the pandemic was less severe. The effects of demand stimulus policies were positive and stronger the less severe the pandemic was while the impact of firm support policies was negative in the regions where the pandemic was more severe. All these effects vanished in the second wave.

## Abstract (plain language summary)

Disasters cause slowdowns in new firm creation that disaster recovery policies may aggravate or alleviate. Our analysis of the effects of the COVID-19 pandemic in Italy reveals that this major disaster resulted in a large drop in new firm creation rates. During the first pandemic wave, the drop was concentrated in the regions where the pandemic was more severe. Examining the effects of the shutdown policies implemented to contain the spread of the disease and the measures designed to protect the economy provides useful guidelines for policymakers. First, we show that shutdown policies inhibit new firm creation. Policymakers can however alleviate this negative effect by implementing less strict measures in the areas not severely affected by the disaster. Second, despite we understand that policymakers need to provide relief to existing firms in the most affected industries to avoid business failures, we indicate they should simultaneously invest in stimulating demand in these industries to sustain also new firm creation.

## Introduction

Severe acute respiratory syndrome coronavirus 2 (SARS-CoV-2) disease (COVID-19) is a public health disaster that started in 2019 in China and quickly spread to the rest of the world, resulting in a pandemic (Hui et al., 2020). Unlike many localized disasters (e.g. natural disasters such as earthquakes, floods or terrorist attacks), which typically imply immediate, but geographically limited direct effects and spillovers at a larger scale, or disasters such as climate change, which affect the global economy but are slow moving, the COVID-19 pandemic rapidly disrupted economic conditions in every area of the globe (Goodell, 2020). Given these devastating consequences, since the first months of 2020, national governments and local authorities of most countries have implemented disaster recovery policies, i.e. policy measures aimed at either dealing with the disruption that the disaster has caused in both community life and the economic system or mitigating disaster’s future hazards (Tierney, 1993). The measures designed to overcome the recessionary effect of the COVID-19 pandemic include rate cuts, banking regulation relaxation, increases in government spending in public health systems and financing (in various forms, such as grants, tax credits, loans) to firms and households (Braunerhjelm, 2022; Shanaev et al., 2020). The policy measures aimed at mitigating the pandemic’s future hazards sought to contain the spread of the disease by introducing closures of places of social gathering (e.g. pubs, restaurants, cinemas, schools and universities), travel restrictions, closures of shops and firms and even (partial and complete) lockdowns (Cohen & Kupferschmidt, 2020).

The COVID-19 pandemic and the related disaster recovery policies have had relevant social consequences. The global spread of the disease and the associated restrictions, combined with contradicting information diffused by mass media and social media, have led people to experience fear, anxiety and depression (Wang et al., 2020). The peculiar characteristics of the COVID-19 disaster and of the related recovery policies and their economic and social consequences have created a unique situation that has no documented equivalent in the entrepreneurship literature (Kuckertz et al., 2020). Thus, it comes as no surprise that entrepreneurship scholars are devoting much effort to studying how entrepreneurs and their ventures have faced the COVID-19 disaster (for reviews of the literature on the effect of COVID-19 on entrepreneurship, see Belitski et al., 2022; Khlystova et al., 2022).

To date, entrepreneurship studies about the pandemic have focused primarily on existing ventures, perhaps because of the compelling need to understand the impact of the COVID-19 disaster on extant economic activities and to leverage this knowledge to design adequate policies to protect entrepreneurship. Yet, scholars have paid limited attention to the effects of the COVID-19 pandemic on the creation of new firms. The few contributions on this topic have investigated the impact of the pandemic on entrepreneurial intentions (Hernández-Sánchez et al., 2020; Ruiz-Rosa et al., 2020) or provided descriptive evidence on new firm creation rates during the pandemic (Duncan et al., 2020). A comprehensive picture of how the COVID-19 pandemic has affected new firm creation is still missing. This is a critical gap; the creation of new firms is a powerful engine of job creation and innovation (Aghion et al., 2009; Audretsch et al., 2006; Klapper et al., 2006) in economic systems, which is of paramount importance to overcome disasters.

This paper takes a step to fill this research gap by addressing two fundamental research questions, namely, (i) how do the effects of the COVID-19 pandemic on new firm creation vary depending on variations in the severity of the pandemic across time and space? (ii) What are the effects of pandemic-related policies, i.e. the disaster recovery measures aimed at either controlling the spread of the disease or protecting the economy from the negative consequences of the pandemic? To address this second research question, we focus on specific policies: the measures that mandated temporary shutdowns of nonessential economic activities to limit the spread of the disease (hereafter: shutdown policies) and the financial support measures aimed at overcoming the pandemic recessionary effects directed toward either consumers (hereafter: demand stimulus policies) or existing firms in specific industries (hereafter: firm support policies).

To answer our research questions, we conduct an exploratory study; we do not anchor on a single theoretical perspective, but we combine theoretical arguments from entrepreneurship literature with theoretical insights and empirical evidence from studies on the COVID-19 pandemic. In particular, we first argue that both pandemic severity and pandemic-related policies alter prospective entrepreneurs’ expectations about the profitability of new firms in the short to medium term. Such altered expectations affect prospective entrepreneurs’ likelihood of creating new businesses, thus ultimately influencing new firm creation. Following this logic, we develop a series of hypotheses about the effects of pandemic severity, pandemic-related policies and the interactions between pandemic severity and policies on new firm creation.

We test our hypotheses using unique data on Italy from January to December 2020, a period that encompasses the first and second waves of the COVID-19 pandemic in the country. This is an ideal context for our work for several reasons. First, in the period under scrutiny, the COVID-19 pandemic has not hit Italy uniformly; the first wave was much more severe in the most developed areas of the country (i.e. the northern regions), where new firm creation is normally higher than in the rest of the country, while during the second wave the pandemic seriously affected southern regions as well. Second, despite geographical differences in the pandemic severity, the type of policies implemented by the Italian government during the first wave were similar across Italian regions, yet differed quite substantially across industries. In contrast, during the second wave, higher regional variation in the pandemic-related policies was detected. Third, the shutdown policies implemented in Italy during the first wave were the most incisive ones, apart from those implemented in China; thus, it is highly interesting to study their effects.

Through this analysis, we contribute to disaster research by providing a better understanding of the effects on new firm creation of a disaster of unprecedented proportions and the associated disaster recovery policies. Furthermore, we shed light on the effect of disaster experience on individuals’ behaviours, by investigating whether prospective entrepreneurs who experienced the first wave of the COVID-19 pandemic adopted different new firm creation behaviours during the second wave.

The paper is structured as follows. In Sect. 2, we first provide a short review of the studies that have investigated the relationship between disasters and entrepreneurial activity. Then, we discuss the effects of pandemic severity and pandemic-related policies on new firm creation and formulate a set of hypotheses. Section 3 briefly describes the evolution of the COVID-19 pandemic in Italy and the pandemic-related policies approved by the Italian government. Section 4 introduces the data sources and provides preliminary univariate analyses. Section 5 describes the econometric specification and the variables included in regression models for the multivariate analyses. The results of the multivariate analyses are outlined in Section 6. Section 7 discusses how the key findings of the study contribute to the academic literature, while the concluding section highlights the limitations of the study, proposes future research avenues and discusses the policy implications of the findings.

## Conceptual framework

### Disasters and entrepreneurial activity

Disasters (or extreme events) are “acute collectively experienced events” that result in a “catastrophic depletion of resources” (Kaniasty & Norris, 1993: 396) and threaten “the lives, economies, and wellbeing of those they impact” (Williams & Shepherd, 2016: 2069). Over the past two decades, disaster research has proliferated and widened the investigated topics (for a systematic review, see Wolbers et al., 2021); nevertheless, there are still some areas of research that deserve further attention.

So far, disaster literature has mainly focused on relief aid management, risk management and economic consequences after the occurrence of disasters, usually adopting a macroeconomic perspective, while it has only tangentially investigated the relationship between disasters and entrepreneurial decisions and actions (for a literature review on disasters and entrepreneurship, see Galbraith & Stiles, 2006).

More specifically, disaster research has mainly focused on the role of entrepreneurship in the aftermath of a disaster: entrepreneurial activity can help the recovery of the economies and communities affected by disasters (Chamlee-Wright & Storr, 2008; Williams & Vorley, 2014), and new firm creation contributes to the psychological functioning of focal entrepreneurs (Williams & Shepherd, 2016). Conversely, the impact of disasters on entrepreneurship is still poorly understood. Some studies have devoted attention to the effect on entrepreneurial intentions of the occurrence and severity of several types of disasters (Brück et al., 2011; Bustamante et al., 2020; Monllor & Murphy, 2017), including the COVID-19 pandemic (Hernández-Sánchez et al., 2020; Ruiz-Rosa et al., 2020), but they did not find conclusive evidence. The impact of disasters on new firm creation is even less understood. To the best of our knowledge, only a recent paper by Edobor and Marshall (2021) has examined the effects of different types of disasters on the creation of new firms, and it has found very little evidence supporting a role for disaster severity (as captured by the dollar amount of disaster damage) in new firm creation.

Knowledge about the impact of disaster recovery policies on entrepreneurship is limited as well. Disaster research has recognized that disaster recovery policies might retard entrepreneurship by crowding out new firms or posing an excessive burden on entrepreneurs, thereby slowing recovery efforts (Chamlee-Wright & Storr, 2008), but no evidence in favour of these arguments has been found.

This brief literature review clearly reveals a need for better understanding the impact of disasters on new firm creation by distinguishing the effects of disaster severity and the disaster recovery policies implemented. In the following, we contribute to improve this understanding.

### Hypotheses

In this section, we discuss the impact on new firm creation of both the severity of the COVID-19 pandemic and the pandemic-related policy measures under scrutiny. We start from the premise shared by the entrepreneurship literature that new firms are created when prospective entrepreneurs who identify business opportunities expect the returns from exploiting these opportunities in new firms to be greater than the associated opportunity costs (Campbell, 1992). Thus, before launching new firms, prospective entrepreneurs assess new firms’ expected profits in the short-medium term. Disasters drive these expectations and thus alter prospective entrepreneurs’ likelihood of launching new businesses (Brück et al., 2011) and ultimately affect new firm creation in the regions affected by the disaster.

We expect that the severity of the COVID-19 pandemic in a region has negative effects on new firm creation in that region because it reduces new firms’ expected profits. First, pandemic severity has negative effects on the expected sales of many products and services. In the regions more affected by the pandemic, consumers are more likely to seek to reduce their risk of exposure to the virus; thus, they, at least temporarily, decrease demand for products and services whose purchase or consumption involves close contact with others (del Rio-Chanona et al., 2020). At the same time, pandemic severity increases expected production costs. The more severe the pandemic is in a region, the more reduced the labour supply in that region due to mortality and morbidity caused by either infection or the need to care for affected family members (McKibbin & Fernando, 2021). Hence, prospective entrepreneurs may anticipate that in regions where the pandemic is more severe, the productivity of local employees is lower, and the labour search costs incurred by local firms are higher, as these firms may need to look for labour from regions that are less affected by the pandemic. Likewise, prospective entrepreneurs may anticipate that morbidity and mortality in regions more severely affected by the pandemic will result in productivity losses and a smaller workforce for the suppliers of their new firms and transport firms. The reduced productivity of suppliers and transport firms may engender delays in service provision, shortages of materials and delays in material delivery for prospective entrepreneurs’ new firms. In addition, the more severe the pandemic is in a given region, the greater the subjective uncertainty about firms’ future sales growth rates (Altig et al., 2020). This greater uncertainty may have two additional negative effects on new firm creation. As uncertainty about future earnings increases the cost of capital for firms (Hribar & Jenkins, 2004), prospective entrepreneurs in the regions hardest hit by the pandemic may experience more severe financial constraints that may inhibit new firm creation. At the same time, greater uncertainty raises prospective entrepreneurs’ fear of failing, thus discouraging new firm creation (Brück et al., 2011). All these arguments lead to the following hypothesis.^1^

**Fig. 1:**
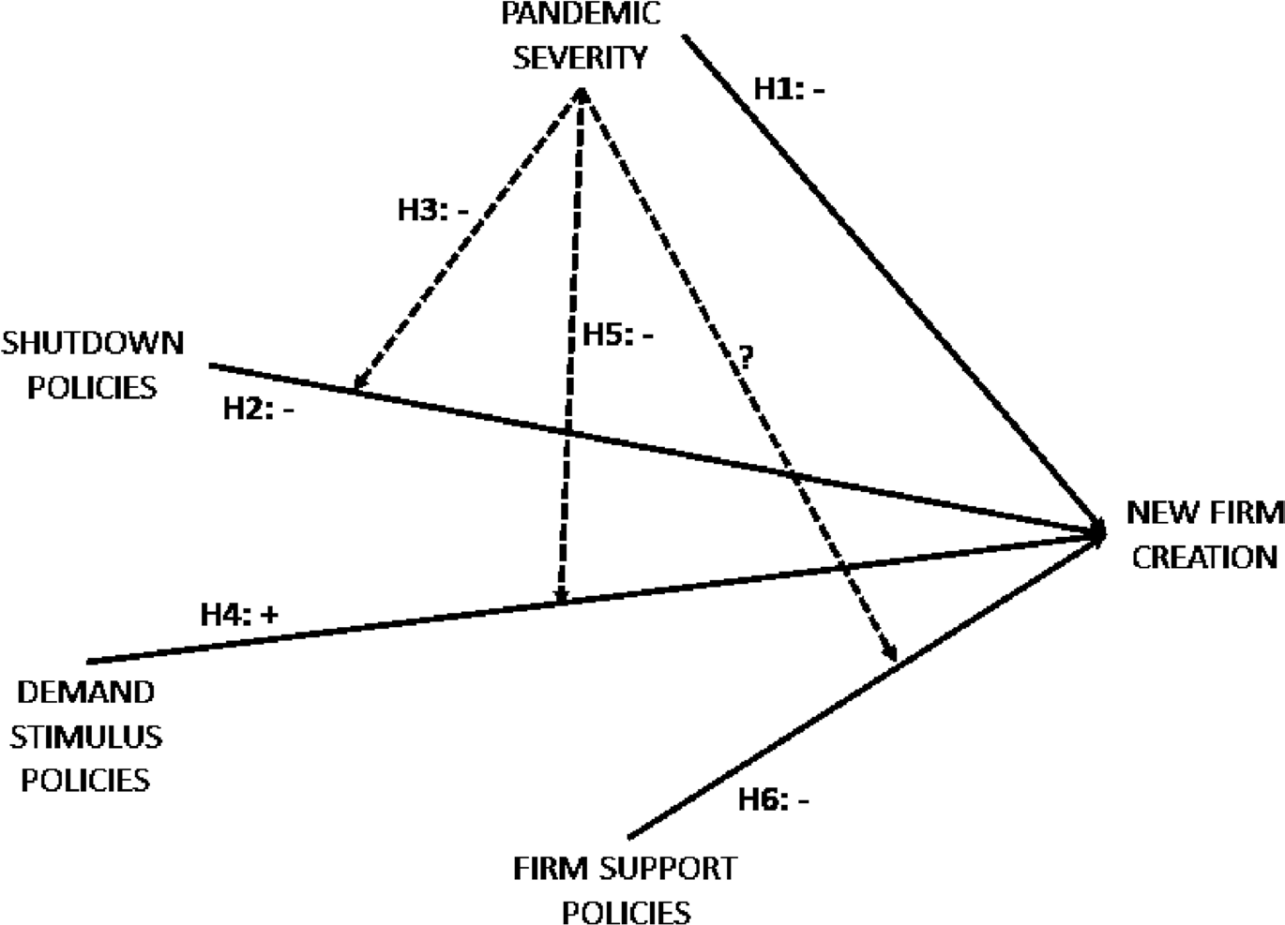
Synthesis of the six hypotheses formulated. The solid lines indicate the direct effects on new firm creation of the variables under scrutiny, while the dotted lines indicate the interactions between pandemic severity and the policy variables

- H1: *Pandemic severity in a given region negatively affects new firm creation in that region*
  We now turn attention to the effects of shutdown policies, which, as we mentioned above, mandate temporary shutdowns of specific nonessential economic activities to control the spread of the disease. We argue that such policies reduce new firms’ expected profits. In several industries (e.g. retail, restaurants, entertainment), shutdown policies result in a drop in revenue expectations because consumers cannot reach the places where products/services are typically sold or consumed (Baldwin & Weder di Mauro, 2020). The negative effects of shutdown policies on expected revenues are particularly severe for firms whose consumption is seasonal, as the sales lost during the shutdown are impossible to recoup after the shutdown has ended.^2^ Although consumers in some industries might change their behaviour and resort to online buying and consumption, the increase in online purchases is unlikely to compensate for the drop in physical sales and consumption, as data on transactions in the first months of the pandemic indicate (Andersen et al., 2020). In spite of this reduction in expected revenues, during shutdowns, firms in nonessential industries are still liable for their continuing fixed costs, such as rent, logistics and storage fees (Lu et al., 2020), which further erode expected profits. Moreover, when workers are not allowed to reach the workplace, expected labour productivity may be lower. Indeed, while some workers experience minor disruptions when switching to their home office, for many workers (e.g. those who require peculiar equipment to do their job), working from home means they cannot perform all their tasks (Adams-Prassl et al., 2020). Finally, in several industries, shutdown policies are expected to increase supply costs. For instance, during shutdowns, firms are likely to incur costly wastages of perishable production inputs if such inputs are not processed promptly after being procured.
  In line with these arguments, we claim that the effect of shutdown policies on new firm creation is negative, and we formulate the following hypothesis.
- H2: *Shutdown policies in a given industry and region negatively affect new firm creation in that industry and region.*
  We expect this negative effect of shutdown policies on new firm creation to be greater in regions where the pandemic is more severe. As mentioned above, both pandemic severity and shutdown policies are expected to decrease new firms’ expected profits. In regions with greater pandemic severity, many prospective entrepreneurs have negative expectations about future profits; hence, they will refrain from creating new firms also in the absence of shutdown policies. Therefore, the additional reduction of expected profits engendered by the implementation of shutdown policies has negligible effects on new firm creation in these regions. Conversely, in regions where pandemic severity is lower, shutdown policies may hinder new firm creation by many prospective entrepreneurs who would not refrain from creating new firms in the absence of such policies. Following these arguments, we conclude that the negative effects of shutdown policies predicted by H2 are less evident in regions where pandemic severity is greater than in the regions less affected by the pandemic.
  We now propose an additional argument in favour of the reduced negative effect of shutdown policies in regions with greater pandemic severity. As shutdown policies are implemented to limit the spread of the disease, prospective entrepreneurs in these regions might expect that the temporary closure of economic activities will help in containing the contagion, thus reducing pandemic severity in the near future and significantly improving the disaster situation. Such expectations of noticeable future reductions in pandemic severity may positively affect new firms’ expected profits and, to some extent, counterbalance the negative effects of shutdown policies on expected profits. Conversely, in regions where pandemic severity is lower, shutdown policies are probably expected to result in less significant improvements in the disaster situation. These expectations of future unremarkable reductions in pandemic severity after implementing shutdown policies may have negligible positive effects on new firms’ expected profits that are thus unable to compensate for the negative effects of the shutdown. Following these arguments, we claim that the negative effects of shutdown policies on firm creation will be weaker in the regions more affected by the pandemic than in the less affected regions. Hypothesis 3 follows.
- H3: The negative relation between shutdown policies and new firm creation in a given industry and region is weaker the greater the pandemic severity in the region.
  Let us now discuss the effects on new firm creation of policy measures aimed at protecting the economic system by funding specific industries hit by the pandemic. These policies vary depending on their direct beneficiaries, which may be either consumers or existing firms. We first consider demand stimulus policies having consumers as direct beneficiaries. These policies are aimed at stimulating the demand for specific nonessential products or services, e.g. by providing consumers with vouchers to purchase these products/services or granting them tax credits after purchase. Demand stimulus policies may positively affect the expectations about new firms’ short-term profits. As tax rebates and, in particular, shopping vouchers tend to have some positive effects on consumers’ spending (e.g. Hsieh et al., 2010; Kan et al., 2017), prospective entrepreneurs may anticipate that the demand in the industries supported by demand stimulus policies will increase in the near future. Hence, prospective entrepreneurs will be more likely to create new firms in these industries. Based on these arguments, we formulate the following hypothesis.
- H4: Demand stimulus policies in a given industry positively affect new firm creation in that industry
  The positive effects on new firm creation of demand stimulus policies are likely to depend on pandemic severity. In regions with greater pandemic severity, individuals experience stronger emotions of fear and anxiety (Le & Nguyen, 2021). Such negative emotions create irregular and often irrational consumer behaviours (Loxton et al., 2020). Prospective entrepreneurs may expect that consumers who do not have entirely rational behaviours may be less responsive than rational consumers to the stimuli of policies aimed at increasing demand for nonessential products/services; hence, demand stimulus policies are unlikely to significantly increase consumers’ spending. Conversely, in regions where pandemic severity is lower, individuals experience less intense negative emotions that are less likely to generate irrational consumer behaviours. Hence, prospective entrepreneurs might expect that policy stimuli will have stronger effects in these regions. Following these arguments, we claim that pandemic severity reduces the positive effect of demand stimulus policies on new firm creation. Hypothesis 5 follows.
- H5: The positive relation between demand stimulus policies in a given industry and new firm creation in that industry is weaker the greater the pandemic severity in the region
  Other policy measures aimed at protecting the economic system are those having existing firms in specific industries as direct beneficiaries. Firm support policies encompass measures such as grants to cover their expenses or to make investments, relief funds aimed at compensating for missed revenues, tax exemptions and temporary suspensions of fee payments. As firm support policies target only the firms that were active at the outbreak of the pandemic, they likely have negative effects on new firm creation. On the one hand, firm support policies engender cost advantages for existing firms with respect to prospective entrants that cannot benefit from these measures. As cost advantages of existing firms serve as important barriers to entry (e.g. Bain, 1956; Porter, 1980), prospective entrepreneurs may be discouraged from entering the industries targeted by firm support policies. On the other hand, firm support policies probably reduce existing firms’ failure rates, thus reducing the amount of resources freed by exited firms and, as a consequence, the probability that new firms are created in the industry to take advantage of these resources. These arguments lead to the following hypothesis.
- H6: Firm support policies in a given industry negatively affect new firm creation in that industry
  Pandemic severity may also affect the negative relationship between new firm creation and firm support policies in given industries, even though this moderating effect is hard to predict. On the one hand, we expect that the negative effect of firm support policies on the creation of new firms will be weaker the greater the pandemic severity. In line with the arguments leading to H1 and H3, in the regions with greater pandemic severity, many prospective entrepreneurs have negative expectations about future profits because of the pandemic. Thus, they will refrain from creating new firms, regardless of firm support policies. Conversely, in regions that are less hit by the pandemic, providing existing firms with cost advantages is likely to reduce the number of prospective entrepreneurs willing to create new firms. On the other hand, we might claim that the negative effect of firm support policies on new firm creation is more intense as pandemic severity increases. In the regions with greater pandemic severity, the probability of failure of existing firms is very high (Amankwah-Amoah et al., 2021); hence, firm support policies may contribute to reducing existing firms’ allegedly high failure rates. Conversely, firm support policies are less likely to reduce existing firms’ failure rates in the least severely affected regions, where existing firms are probably less likely to fail.
  As opposing forces are at work, we do not formulate any hypothesis on the moderating effect of pandemic severity on the relationship between new firm creation and firm support policies.
  Our six hypotheses are synthesized in Fig. 1.

## The Italian context during the COVID-19 pandemic

### The spread of COVID-19 in Italy and the policies taken to control the spread of the disease

In Italy, the first cases of COVID-19 were identified at the end of January 2020. In the following weeks, several clusters of cases were progressively detected in Northern Italy, and the first deaths of people infected by SARS-CoV-2 occurred. To minimize the likelihood that people who were not infected came into contact with infected people, on February 22, the Italian government identified 11 municipalities located in two northern regions (Lombardy and Veneto) where the number of infected people was sizable and established a 2-week total lockdown for these areas. In these municipalities, schools and shops—except those selling essential items (e.g. grocery stores, food stores, and pharmacies)—were closed, public gatherings were prohibited, and there were significant restrictions on people’s mobility, with residents forced to stay within their municipality.

As the numbers of infections, hospitalized people and deaths quickly rose in many areas of Italy, on March 9, the Italian government announced a 2-week total national lockdown. In the following weeks, the length of the national lockdown was progressively extended until May 3. During the national lockdown, all firms, except those operating in so-called *essential industries*^3^ listed on the Italian Prime Minister’s decrees of March 22 and 25, had to shut down.^4^ When the national lockdown ended, firms in most industries progressively restarted their operations. Since June 1, the Italian productive system began to operate almost normally, but all economic activities had to implement precautions mandated by the government and local authorities to suppress contagions (e.g. guaranteeing social distancing of workers and customers).

The shutdown policies described above contributed to the decline in the number of COVID-19 cases, hospitalized people and deaths. Nevertheless, starting in August 2020, Italy witnessed a new rise in detected COVID-19 cases and severe infections. In October, as it was clear that Italy had entered a second wave of the pandemic, the Italian government reintroduced strict rules to limit the spread of the disease: gatherings of people were forbidden; gyms, swimming pools, theatres and cinemas were closed; and bars and restaurants had limited working hours. Quite interestingly, during the second wave of the pandemic, the Italian government changed its approach to the implementation of shutdown policies. First, no total lockdowns were implemented. Second, the government did not impose the same restrictions in the whole of Italian territory, as it did during the first wave. Since November 6, Italian regions were classified into three groups—yellow, orange and red—corresponding to three risk scenarios (from low to high risk of contagion, respectively), for which specific restrictive measures were implemented. The classification of each group was based on ordinances issued every week by the Ministry of Health.^5^

**Fig. 2:**
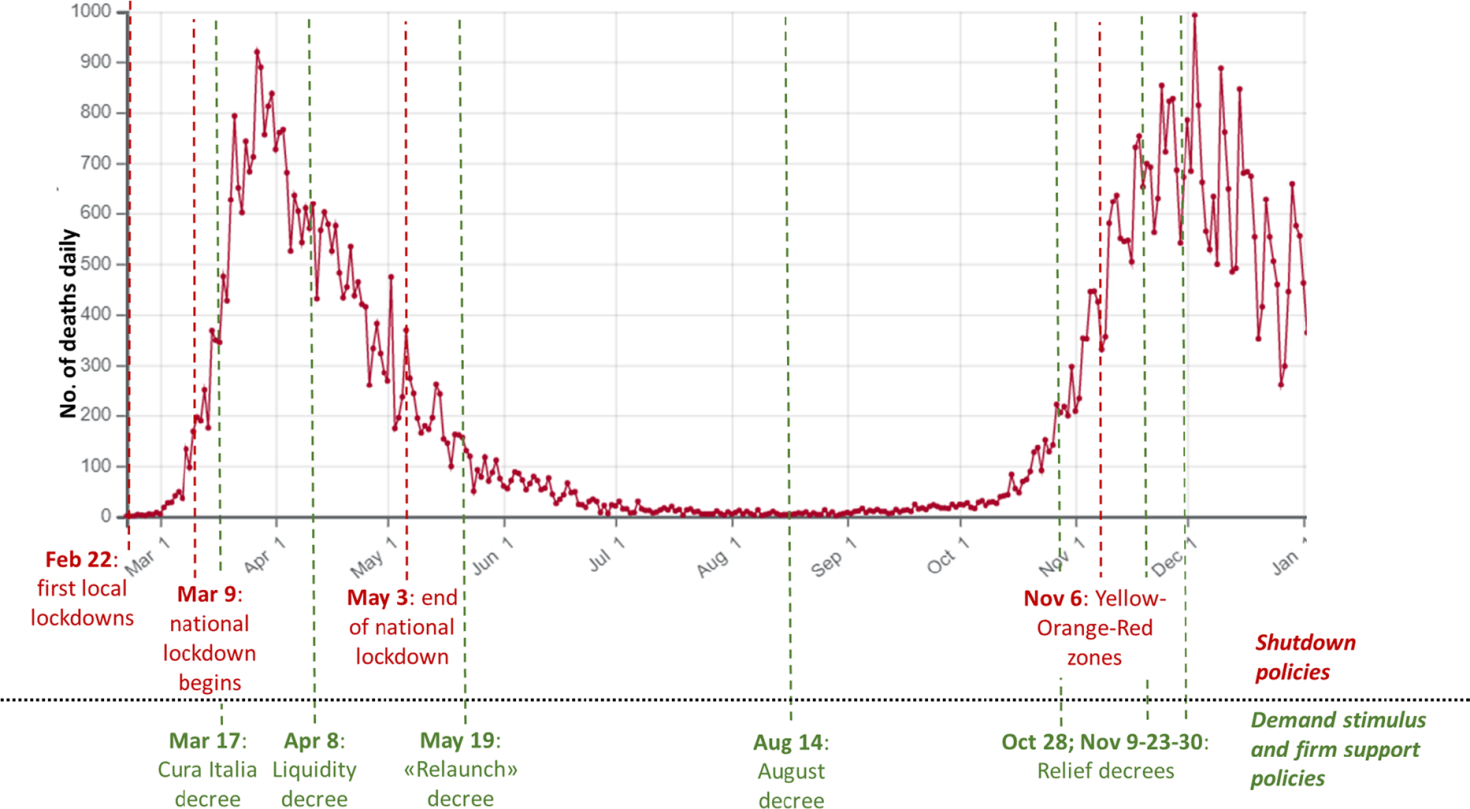
Timeline of the COVID-19 pandemic in Italy till the end of 2020: daily deaths and key policy measures. The graph reports the evolution of the number of daily COVID-19 deaths since the last days of February 2020 till the end of December 2020. The red dates and dotted lines capture the key shutdown policies implemented by the Italian government; the green dates and dotted lines capture the approval by the Italian government of the key demand stimulus and firm support policies

The evolution of the COVID-19 pandemic in Italy until the end of 2020 and the shutdown policies implemented by the Italian government are shown in Fig. 2.

### The policies approved by the Italian government to protect the economy

Although the shutdown policies imposed by the Italian government had the positive effect of limiting the spread of the disease, temporarily halting commercial, production and service activities had severe impacts on the national economy. Hence, since mid-March 2020, the Italian government started developing economic recovery plans and progressively approved tax and expenditure measures intended to support firms generally and those operating in specific industries.^6^ All firm support policies were aimed at sustaining already existing firms.^7^ Moreover, both existing and new firms could obtain indirect advantages from demand stimulus policies whose direct beneficiaries were consumers. The key firm support and demand stimulus policies approved by the Italian government to face the economic crisis caused by the pandemic were reported in the eight decree laws reported in Fig. 2. Appendix Table 7 reports the firm support and demand stimulus policies targeting specific industries and included in these eight decree laws of the Italian government.

## Data and descriptive evidence on the Italian case

To examine how the severity of the COVID-19 pandemic and the pandemic-related policies designed by the Italian government affected new firm creation in Italy in 2020, we use data collected from several sources.

Data on new firm creation were extracted from the Infocamere Business Register, managed by the Italian Chambers of Commerce, which gathers information on all new firms registered in Italy. For each firm, the Business Register stores information on the registration date, the legal form, the industry of operation and the location declared at the registration date. Using the Business Register, we gathered monthly data on the number of new limited liability companies created (i.e. registered at the Chambers of Commerce) in each Italian province (corresponding to the NUTS 3 level in the Eurostat classification of regions) and industry (NACE 2-digit level, in line with Klapper & Love, 2011) in 2020. For the sake of comparison, we gathered these data for 2018 and 2019.

Data on the variation in pandemic severity across time and space were extracted from the COVID-19 database created at the end of February 2020 and updated daily by the Italian Civil Protection Department.^8^ This database contains data on the spread of COVID-19 across the country; as most data are available only at the regional level, in the following, we use the Italian region (NUTS 2) as the geographical unit of analysis. This unit of analysis is particularly appropriate to study the effects of the pandemic-related policies under scrutiny. Although firm support and demand stimulus policies targeted the Italian territory as a whole, shutdown policies differed across regions (with the only exceptions of the initial lockdown measures that focused on specific municipalities).

Information on the pandemic-related policies under scrutiny was retrieved from the website of the Presidency of the Council of Ministers,^9^ which reports the descriptions of all the measures related to the COVID-19 emergency approved by the Italian government.

**Fig. 3:**
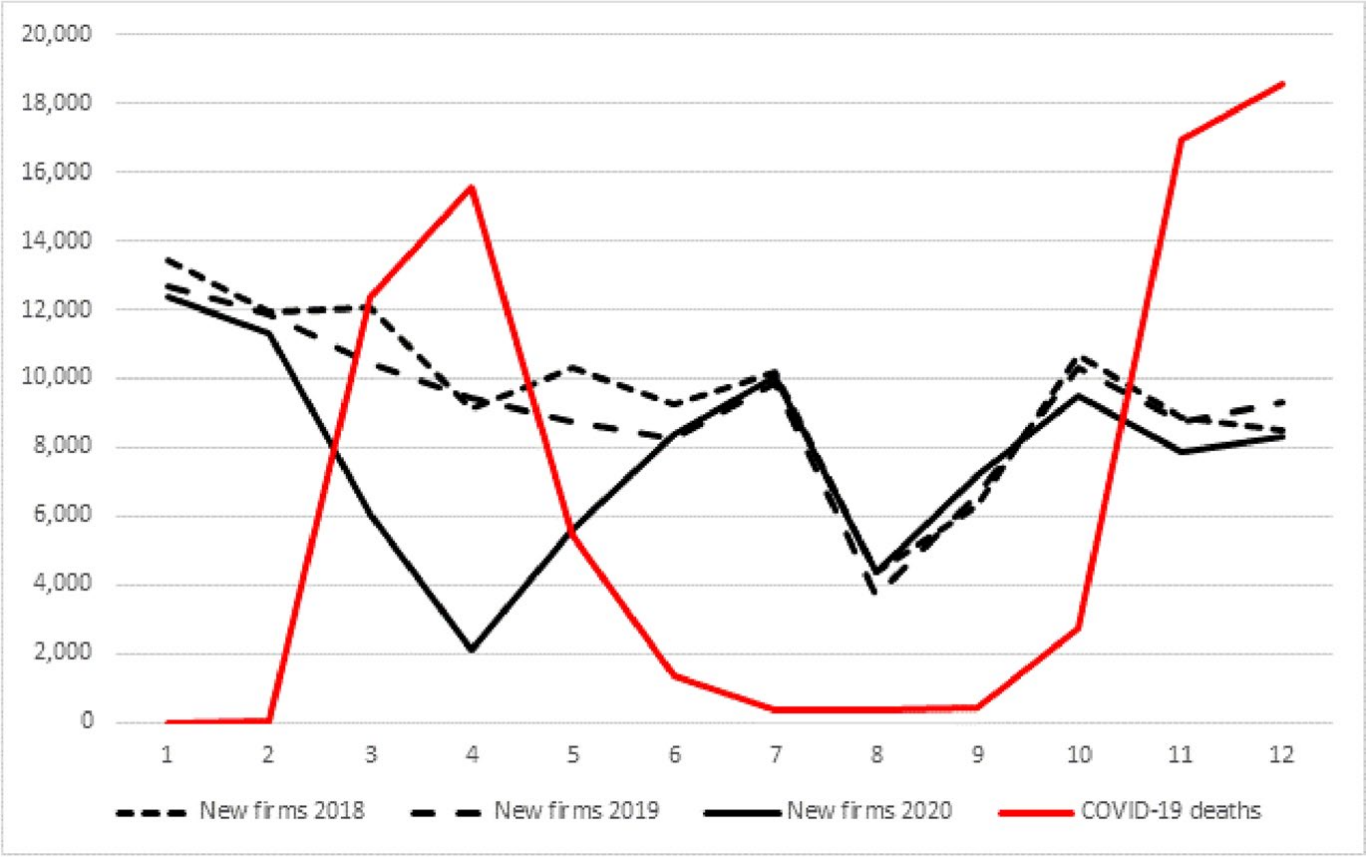
New limited liability companies created in Italy in 2018, 2019 and 2020 and number of COVID-19 infected people who died in Italy in 2020

First, we use the data extracted from the Infocamere Business Register to explore the evolution in the number of new limited liability companies registered in Italy before and during the COVID-19 pandemic. Figure 3 reveals that new firm creation in Italy is characterized by a marked seasonality. As is evident from data from 2018 and 2019, the creation of new firms is lively in the first months of the year, while it reaches its minimum in August, when most Italian people are on holiday. Moreover, Fig. 3 shows that Italy experienced a 15% drop in new firm creation in 2020, with 93,103 new limited liability companies created vs. 110,049 created in 2019. As Fig. 3 shows, the drop was larger between March and May, which, as we mentioned above, was the peak period of the first pandemic wave and when the Italian government implemented the total national lockdown. While in both the January–February and June–September periods, new firm creation in 2020 is in line with the figures of 2019 and 2018, in the March–May period, the average monthly drop between 2019 and 2020 was 52%, with the maximum drop achieved in April (− 78%). This temporary slowdown in new firm creation would not have been a concern if the number of new firms created in 2020 between June and September had been significantly more than the new firms created in the same months of 2019 and 2018, thus suggesting that during the first pandemic wave, firm creation was merely delayed. Unfortunately, Fig. 3 shows that in 2020, in every month of the June–September period, the number of new firms created was slight but not significantly higher than the corresponding numbers in 2019 and 2018, thus indicating that the new firms that Italy “lost” between March 2020 and May 2020 were not created in the following months. In the October-December period in 2020, which corresponds to the second pandemic wave (as the red line in Fig. 3 indicates), Italy experienced a new drop in new firm creation (− 9%) that was much smaller than the drop that had occurred in the first wave.

Let us now focus on regional variations in pandemic severity and new firm creation. Table 1 shows that in 2020, Italian regions differed sharply with regard to pandemic severity. The last column of Table 1 reports the number of people infected by SARS-CoV-2 who died in 2020 per 10,000 inhabitants, revealing that the pandemic was particularly severe in the northern regions, while the southern regions were barely affected. Conversely, as the second column of Table 1 indicates, in 2020, in *all* Italian regions, there was a drop in new firm creation of between 13 and 20% (the decrease was less than − 10% only in Aosta Valley, a very small region where few firms are created). Evidence from this univariate analysis does not suggest a clear link between pandemic severity and new firm creation at the regional level. The general drop in new firm creation across Italian regions can, however, be attributed to the national lockdown implemented during the first pandemic wave. We refer to the multivariate analysis presented in Section 6 for more conclusive evidence on the distinct effects of pandemic severity and shutdown policies on new firm creation.

**Table 1:** Percentage variation in the number of new limited liability companies created in 2020 and 2019 and overall pandemic severity in Italian regions

	2019–2020 variation	COVID-19 deaths per 10,000 inhabitants
*North*		
Aosta Valley	− 6.0%	30.20
Emilia-Romagna	− 15.0%	17.32
Friuli-Venezia Giulia	− 16.7%	13.56
Liguria	− 18.3%	18.73
Lombardy	− 13.4%	24.86
Piedmont	− 14.1%	18.25
Trentino-South Tyrol	− 13.0%	15.64
Veneto	− 11.8%	13.32
*Centre*		
Latium	− 19.4%	6.43
Marche	− 18.1%	10.35
Tuscany	− 17.1%	9.87
Umbria	− 15.8%	7.09
*South and islands*		
Abruzzo	− 16.4%	9.29
Apulia	− 15.7%	6.17
Basilicata	− 18.6%	4.60
Calabria	− 14.8%	2.45
Campania	− 13.4%	4.92
Molise	− 16.7%	6.32
Sardinia	− 16.5%	4.58
Sicily	− 16.0%	4.85

We now turn our attention to cross-industry differences in new firm creation. Table 2 reports the 2019–2020 percentage variation in the number of new limited liability companies across the 30 industries with the highest new firm creation rates during the 2019–2020 period. Industries are listed in increasing order of percentage variation. Several industries experienced dramatic drops in the number of newly created firms. Sports activities and food and beverage service activities (where the number of new limited liability companies declined by 39% and 36% between 2019 and 2020, respectively) are a case in point. Key players in these industries are gyms, indoor recreation sites, restaurants, cafeterias and bars, which since February 2020 have been subjected to thorough restrictions, including long periods of closure, reduced working hours and limitations in seating capacity to maintain social distancing. These restrictions have likely negatively affected the profit expectations of prospective entrepreneurs, thus reducing their likelihood of establishing new firms and the actual numbers of new firms created in this industry. Interestingly, Table 2 reveals that (few) industries experienced increases in the number of newly created firms. This is the case for the education sector (where the number of newly created firms increased by 15% between 2019 and 2020). The COVID-19 pandemic has stimulated innovation in this sector; innovative approaches in support of education and training have been implemented, thus generating new business opportunities in this industry.

**Table 2:** Percentage variation in the number of new limited liability companies created in Italy in 2020 and 2019 in the top 30 industries by new firm creations

Industry (NACE code)	2019–2020 variation
Sports activities and amusement and recreation activities (R 93)	− 39.49%
Food and beverage service activities (I 56)	− 36.43%
Manufacture of fabricated metal products, except machinery and equipment (C 25)	− 28.00%
Wholesale and retail trade and repair of motor vehicles and motorcycles (G 45)	− 23.43%
Other personal service activities (S 96)	− 23.23%
Accommodation (I 55)	− 23.14%
Manufacture of machinery and equipment (C 28)	− 21.40%
Warehousing and support activities for transportation (H 52)	− 19.11%
Electricity, gas, steam and air conditioning supply (D 35)	− 18.35%
Repair and installation of machinery and equipment (C 33)	− 14.71%
Office administrative, office support and other business support activities (N 82)	− 13.81%
Agriculture, forestry and fishing (A 01)	− 13.35%
Manufacture of wearing apparel (C 14)	− 9.24%
Building completion and finishing (F 43)	− 8.60%
Real estate activities (L 68)	− 6.76%
Wholesale trade, except of motor vehicles and motorcycles (G 46)	− 6.44%
Rental and leasing activities (N 77)	− 6.29%
Manufacture of food products (C 10)	− 6.22%
Retail trade, except of motor vehicles and motorcycles (G 47)	− 5.35%
Advertising and market research (M 73)	− 3.79%
Scientific research and development (M 72)	− 1.83%
Activities of head offices (M 70)	0.72%
Other professional, scientific and technical activities (M 74)	1.06%
Construction of buildings (F 41)	2.41%
Computer programming, consultancy and related activities (J 62)	7.53%
Services to buildings and landscape activities (N 81)	8.14%
Information service activities (J 63)	8.26%
Financial service activities, except insurance and pension funding (K 64)	9.62%
Architectural and engineering activities; technical testing and analysis (M 71)	11.46%
Education (P 85)	15.23%

## Econometric models

We explore the effects of pandemic severity, shutdown policies and firm support and demand stimulus policies approved by the Italian government on new firm creation in Italy in 2020 through a series of econometric models. We use the following specification:

1 $$NewFirmCreationDensityijt\hspace{0.17em}=\hspace{0.17em}\alpha i\hspace{0.17em}+\hspace{0.17em}\beta 1\hspace{0.17em}\times \hspace{0.17em}COVID19Severityit\hspace{0.17em}+\hspace{0.17em}\beta 2\hspace{0.17em}\times \hspace{0.17em}Policiesijt\hspace{0.17em}+\hspace{0.17em}\beta 3\hspace{0.17em}\times \hspace{0.17em}COVID19Severityit\hspace{0.17em}\times \hspace{0.17em}Policiesijt\hspace{0.17em}+\hspace{0.17em}\beta 4\hspace{0.17em}\times \hspace{0.17em}Controlsijt\hspace{0.17em}+\hspace{0.17em}\varepsilon ijt$$

with *i* denoting the Italian region, *j* the industry and *t* the month.

The dependent variable *NewFirmCreationDensity*_*ijt*_ is the number of limited liability companies registered in region *i*, industry *j*, month *t* per 100,000 inhabitants (for a similar measure, see Klapper & Love, 2011). Given the panel data nature of the dataset, we run fixed effects panel regression models where the cross-sectional unit is the region-industry pair, and the time series dimension is defined by the month. Fixed effects regression models allow us to control for all time-invariant unobserved heterogeneity (Wooldridge, 2002) among region-industry pairs in our sample.^10^ Overall, we observe 1701 region-industry pairs (21 regions^11^ × 81 industries) over 12 months, totalling 24,414 observations. We chose the region-industry pair as the cross-sectional unit of analysis because pandemic severity differed across regions, while policies approved by the Italian government to either control the spread of the disease or protect the economy differed by industries and regions.

The first explanatory variable in EQ. (1) — *COVID19Severity*_*it*_ — accounts for pandemic severity. It is defined as the number of people infected with COVID-19 who died in region *i* in month *t* per 1000 inhabitants in the region. It is worth acknowledging that *COVID19Severity*_*it*_ underestimates the overall severity of the COVID-19 pandemic between March 2020 and May 2020. Indeed, in this peak period, large-scale population testing was not carried out due to the limited number of testing centres, the shortage of test kits and the long waiting times for tests; hence, numerous infected individuals died untested and untreated.^12^ To check that the findings are not driven by this measurement error, we replicate our analyses using alternative measures for pandemic severity; such measures are based on the number of active COVID-19 infected cases, hospitalized infected people and intensive care unit patients (see Sect. (6.2 for a discussion of these robustness checks).

The vector *Policies*_*ijt*_ includes variables related to the policies put in place by the Italian government in response to the COVID-19 pandemic. *ShutdownPolicies*_*ijt*_ captures temporary shutdowns of firm activities that the Italian government imposed on some industries. It is the percentage of days in month *t* when the firms located in region *i* and operating in industry *j* were forced to shut down their activities to contain the spread of COVID-19. *DemandStimulusPolicies*_*jt*_ captures the financing policy measures having consumers (individuals or firms) as direct beneficiaries and aimed at stimulating their demand for products/services of industry *j*. *FirmSupportPolicies*_*jt*_ captures the financing policy measures having the firms operating in industry *j* as direct beneficiaries and aimed at either reducing their costs or compensating for missed revenues. *DemandStimulusPolicies*_*jt*_ and *FirmSupportPolicies*_*jt*_ are computed as the amount of money allocated until the end of month *t* to stimulate demand and to sustain existing firms, respectively.

The vector *Controls*_*ijt*_ includes two control variables. *PriorFirmCreationDensity*_*ijt*_ is the average value between 2018 and 2019 of the number of newly registered limited liability companies in region *i*, industry *j* and month *t* per 100,000 inhabitants. This control is aimed at correcting for seasonality effects in new firm creation. *TestsDensity*_*it*_ is the number of COVID-19 tests performed in month *t* per inhabitant in region *i*. This control is included to alleviate the biases that could result from the above-described measurement errors of *COVID19Severity*_*it*_ in the pandemic peak period between March and May. As we run a fixed effects model, the vector *Controls*_*ijt*_ does not include any time-invariant control.

Table 3 reports summary statistics of the variables described thus far and the correlation matrix.

**Table 3:** Summary statistics on regression variables and correlation matrix

	*Variable*	Mean	SD	(1)	(2)	(3)	(4)	(5)	(6)
(1)	*NewFirmCreationDensity*_*ijt*_	0.05	0.14	1.00					
(2)	*COVID19Severity*_*it*_	0.10	0.17	− 0.04	1.00				
(3)	*ShutdownPolicies*_*ijt*_	0.08	0.24	− 0.06	0.21	1.00			
(4)	*DemandStimulusPolicies*_*jt*_	0.08	0.38	0.09	0.00	− 0.02	1.00		
(5)	*FirmSupportPolicies*_*it*_	0.05	0.29	0.22	0.02	0.03	0.14	1.00	
(6)	*PriorFirmCreationDensity*_*ijt*_	0.05	0.14	0.70	− 0.03	0.04	0.22	0.08	1.00
(7)	*TestsDensity*_*it*_	0.04	0.04	0.00	0.53	− 0.06	0.07	0.08	− 0.05

Number of observations: 20,412 (1,701 region-industry pairs observed for 12 months)

## Results

### Main econometric estimates

Tables 4, 5 and 6 report the estimates of the fixed effects regression models based on Eq. (1). The models in Table 4 include only the controls and the measure of pandemic severity; in Table 5, we add the variables on the pandemic-related policies under scrutiny, while in Table 6, we also add the interaction terms between these latter variables and the pandemic severity measure. Each table includes three econometric models: Model 1 is estimated for the whole period under scrutiny (January–December 2020), Model 2 for the first pandemic wave only (January–August) and Model 3 for the second wave (September–December). We separately analyse the two waves of the pandemic because we recognize that fundamental differences exist between the January–August and September–December periods, and these differences may affect the results. As shown in Sect. 4, differences exist in the distribution of the key variables of interest between the two waves.^13^ Moreover, prospective entrepreneurs may have adopted different new firm creation behaviours in the second wave, as they likely benefitted from the experience they acquired with the pandemic during the first wave.

**Table 4:** Results from fixed-effects regression models on the impact of pandemic severity on new firm creation

	Model 1 January–December period	Model 2 January–August period	Model 3 September–December period
Constant	0.019 (0.003) ***	0.033 (0.004) ***	0.006 (0.004)*
*PriorFirmCreationDensity*_*ijt*_	0.477 (0.036)***	0.455 (0.045) ***	0.645 (0.059)***
*TestsDensity*_*it*_	0.183 (0.032)***	− 0.371 (0.085) ***	0.224 (0.080)***
*COVID19Severity*_*it*_	− 0.050 (0.009)***	− 0.088 (0.010)***	− 0.019 (0.017)
No. of observations	20,412	13,608	6,804
No. of region-industry pairs	1,701	1,701	1,701
No. of months	12	8	4
Overall *R*^2^	0.496	0.495	0.520

Robust and clustered standard errors at the region-industry level are in round brackets

^*^Significance at the 10% level; **significance at the 5% level; ***significance at the 1% level

**Table 5:** Results from fixed-effects regression models on the impact of pandemic-related policies on new firm creation

	Model 1 January–December period	Model 2 January–August period	Model 3 September–December period
Constant	0.025 (0.003)***	0.036 (0.004)***	0.006 (0.006)
*PriorFirmCreationDensity*_*ijt*_	0.471 (0.038)***	0.443 (0.048)***	0.645 (0.060)***
*TestsDensity*_*it*_	0.096 (0.030)***	− 0.380 (0.077)***	0.221 (0.082)***
*COVID19Severity*_*it*_	− 0.022 (0.009)**	− 0.039 (0.010)***	− 0.017 (0.016)
*ShutdownPolicies*_*ijt*_	− 0.049 (0.005)***	− 0.051 (0.005)***	− 0.004 (0.013)
*DemandStimulusPolicies*_*jt*_	0.009 (0.004)**	0.014 (0.005)***	0.028 (0.032)
*FirmSupportPolicies*_*it*_	− 0.022 (0.015)	− 0.032 (0.019)*	− 0.039 (0.025)
No. of observations	20,412	13,608	6,804
No. of region-industry pairs	1,701	1,701	1,701
No. of months	12	8	4
Overall *R*^2^	0.488	0.484	0.480

Robust and clustered standard errors at the region-industry level are in round brackets

^*^Significance at the 10% level; **significance at the 5% level; ***significance at the 1% level

**Table 6:** Results from fixed-effects regression models on the interactions between pandemic severity and pandemic-related policies on new firm creation

	Model 1 January–December period	Model 2 January–August period	Model 3 September–December period
Constant	0.024 (0.003)***	0.036 (0.004)***	0.001 (0.008)
*PriorFirmCreationDensity*_*ijt*_	0.470 (0.038)***	0.446 (0.048)***	0.635 (0.059)***
*TestsDensity*_*it*_	0.109 (0.030)***	− 0.383 (0.077)***	0.240 (0.078)***
*COVID19Severity*_*it*_	− 0.023 (0.009)***	− 0.048 (0.010)***	− 0.013 (0.013)
*ShutdownPolicies*_*ijt*_	− 0.058 (0.007)***	− 0.059 (0.006)***	− 0.038 (0.044)
*DemandStimulusPolicies*_*jt*_	0.009 (0.005)**	0.019 (0.005)***	0.034 (0.034)
*FirmSupportPolicies*_*it*_	− 0.013 (0.015)	− 0.015 (0.016)	0.015 (0.040)
*ShutdownPolicies*_*ijt*_ × *COVID19Severity*_*it*_	0.050 (0.029)*	0.071 (0.024)***	0.121 (0.156)
*DemandStimulusPolicies*_*jt*_ × *COVID19Severity*_*it*_	− 0.003 (0.026)	− 0.196 (0.063)***	− 0.047 (0.047)
*FirmSupportPolicies*_*it*_ × *COVID19Severity*_*it*_	− 0.082 (0.030)***	− 0.376 (0.106)***	− 0.072 (0.039)*
No. of observations	20,412	13,608	6804
No. of region-industry pairs	1701	1701	1701
No. of months	12	8	4
Overall *R*^2^	0.489	0.484	0.504

Robust and clustered standard errors at the region-industry level are in round brackets

^*^Significance at the 10% level; **significance at the 5% level; ***significance at the 1% level

Let us briefly discuss the effects of the control variables. In Table 4, the coefficients of the controls are significant in all models. The positive coefficients of *PriorFirmCreationDensity*_*ijt*_ confirm that new firm creation in Italy is characterized by clear seasonality, even during the pandemic. Instead, the coefficient of *TestsDensity*_*it*_ is negative in the first wave and positive in the second wave. This difference probably results from the different diffusion of COVID-19 tests in the two waves; during the first wave, more tests were conducted in the regions more severely hit by the pandemic, while during the second wave, many more tests were available in all Italian regions. Let us now discuss the effects of pandemic severity. In Model 1, the coefficient of *COVID19Severity*_*it*_ is negative and significant and thus indicates that, in line with H1, new firm creation in 2020 was lower in the regions where the pandemic was more severe. The magnitude of the effect of pandemic severity is large: a one standard deviation increase in *COVID19Severity*_*it*_ (0.17) leads to a 0.008 (i.e. 0.050 × 0.17) decrease in *NewFirmCreationDensity*_*ijt*_, which corresponds to a 17% decrease in the average value of *NewFirmCreationDensity*_*ijt*_ in our sample (0.05). Separately analysing the two waves reveals interesting effects. The negative effect of pandemic severity on new firm creation is concentrated in the first wave; the estimates of Model 2 indicate that a one standard deviation increase in *COVID19Severity*_*it*_ leads to a 30% (i.e. 0.17*0.088/0.05) decrease in *NewFirmCreationDensity*_*ijt*_ with respect to its average value in the sample. Conversely, the non-significant coefficient of *COVID19Severity*_*it*_ in Model 3 indicates that in the second wave, pandemic severity did not affect new firm creation in Italy.

The models in Table 5 include the variables related to the shutdown, the demand stimulus and the firm support policies approved in Italy in 2020. In Model 1, the negative and significant coefficient of *ShutdownPolicies*_*ijt*_ indicates that new firm creation in a region-industry was reduced when the firms in that region-industry had to shut down activities for longer periods. Instead, the positive and significant coefficient of *DemandStimulusPolicies*_*jt*_ indicates that when policy measures aimed at stimulating the demand of the products/services sold by firms in a specific industry were approved, prospective entrepreneurs probably anticipated demand increases that generated expectations of greater profits for new firms thus having positive effects on new firm creation in that industry. The effects of both *ShutdownPolicies*_*ijt*_ and *DemandStimulusPolicies*_*jt*_ are of large magnitude. When the value of *ShutdownPolicies*_*ijt*_ switches from 0 (no shutdown restrictions in the region-industry) to 1 (a 1-month shutdown), *NewFirmCreationDensity*_*ijt*_ decreases by 98% (i.e. 0.049/0.05) with respect to its average value in the sample. Instead, a one standard deviation increase in *DemandStimulusPolicies*_*jt*_ (0.38) leads to a 68% (i.e. 0.38 × 0.009/0.05) increase in *NewFirmCreationDensity*_*ijt*_. Interestingly, the coefficient of *FirmSupportPolicies*_*jt*_ is not significant in Model 1.

Models 2 and 3 in Table 5 reveal that while in the first wave of the pandemic, the effects of shutdown and demand stimulus policies on new firm creation were strong, in the second wave, these effects disappeared. Moreover, in Model 2, the coefficient of *FirmSupportPolicies*_*jt*_ is negative and significant at 90%. In summary, the results of the first pandemic wave provide strong support for H2 and H4 and weak support for H6. Conversely, the results of the second wave do not provide support for our hypotheses.

In Table 6, we include the interactive terms between the policy variables and *COVID19Severity*_*it*_. In the estimates on the second pandemic wave (i.e. Model 3), the coefficients of all explanatory variables are not significant (with the only exception of the weakly significant coefficient of *FirmSupportPolicies*_*jt*_ × *COVID19Severity*); therefore, in the following, we discuss only the results on the first wave (i.e. Model 2). In Model 2, all interactive effects are significant at 99%. The positive coefficient of *ShutdownPolicies*_*ijt*_ × *COVID19Severity*_*it*_ indicates that in line with H3, the greater the pandemic severity, the greater the decrease of the negative effect on new firm creation of the shutdown policies implemented in Italy during the first wave of the pandemic. The negative coefficient of *DemandStimulusPolicies*_*jt*_ × *COVID19Severity*_*it*_ suggests that the positive effect of demand stimulus policies was weakened by the pandemic severity, thus providing support for H5. Quite interestingly, the coefficient of the interactive term *FirmSupportPolicies*_*jt*_ × *COVID19Severity*_*it*_ is negative and strongly significant, which implies that firm support policies had a stronger effect on new firm creation in the Italian regions where the pandemic was more severe.

**Fig. 4:**
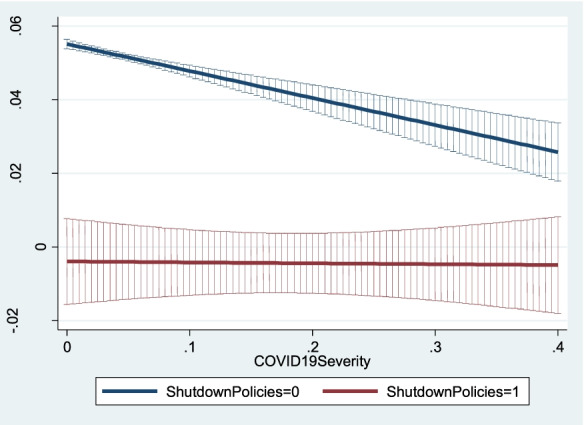
Predicted new firm creation for different levels of shutdown policies as pandemic severity increases

**Fig. 5:**
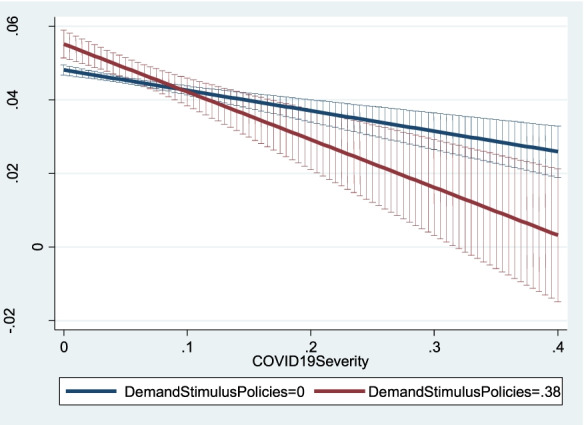
Predicted new firm creation for different levels of demand stimulus policies as pandemic severity increases

**Fig. 6:**
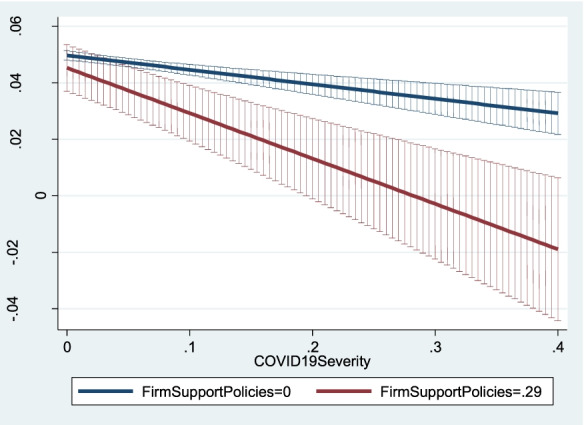
Predicted new firm creation for different levels of firm support policies as pandemic severity increases

To ease the interpretation of the interactive effects, Figs. 4, 5 and 6 show how the predicted values of *NewFirmCreationDensity*_*ijt*_ change (with 95% confidence intervals) as *COVID19Severity*_*it*_ increases for different values of the three policy variables. Predicted values and confidence intervals are computed based on Model 2 in Table 6. Figure 4 indicates that for low values of *COVID19Severity*_*it*_, imposing long shutdowns of firm activities led to a dramatic decrease in new firm creation, as shown by the differences between the blue line (i.e. the predicted value of *NewFirmCreationDensity*_*ijt*_ when *ShutdownPolicies*_*ijt*_ equals 0) and the red line (i.e. the predicted value of *NewFirmCreationDensity*_*ijt*_ when *ShutdownPolicies*_*ijt*_ equals 1). Conversely, as pandemic severity increases, the effect of implementing shutdown policies is less pronounced, as shown by the smaller difference between the blue and red lines for high values of *COVID19Severity*_*it*_. In the regions where the COVID-19 pandemic was more severe, pandemic severity discouraged prospective entrepreneurs from creating new firms so that the additional negative effect on new firm creation engendered by the implementation of shutdown policies was negligible.

Figure 5 shows the predicted values of *NewFirmCreationDensity*_*ijt*_ as *COVID19Severity*_*it*_ increases at different levels of demand stimulus policies. Specifically, the blue line refers to the absence of demand stimulus policies (*DemandStimulusPolicies*_*jt*_ = 0), while the red line corresponds to *DemandStimulusPolicies*_*jt*_ = 0.38 (i.e. the value of its standard deviation in the sample). For low values of *COVID19Severity*_*it*_, the red line lies above the blue line, i.e. the effect of *DemandStimulusPolicies*_*jt*_ is positive and significant. Conversely, in line with H5, as pandemic severity increases, the positive effect of demand stimulus policies becomes weaker, and at high levels of pandemic severity, it is negligible.

Figure 6 shows the relation between *NewFirmCreationDensity*_*ijt*_ and *COVID19Severity*_*it*_ when *FirmSupportPolicies*_*jt*_ equals 0 (the blue line) and when *FirmSupportPolicies*_*jt*_ equals its standard deviation, 0.29 (the red line). For low values of *COVID19Severity*_*it*_, the two lines overlap. However, as pandemic severity increases, new firm creation is significantly lower in the industries that benefited from firm support policies. Greater pandemic severity could have led many incumbents to exit, thus stimulating some prospective entrepreneurs to enter the market by creating new firms to take advantage of the resources freed by exited incumbents, despite the hostile business environment. Nevertheless, this positive effect of incumbents’ exits on new firm creation was probably weakened by firm support policies that may have diminished incumbents’ failure rates in the most severely hit regions.

### Robustness checks and additional analyses

To test the robustness of our results, we conducted three main checks.^14^

First, instead of running fixed effects panel regressions, we ran random effects panel regressions and added different sets of time-invariant control variables, which cannot be included in the fixed effects models. In preliminary estimates, we included industry and region dummy variables in the list of controls and obtained results consistent with those discussed above. In additional analyses, beyond the industry and region dummies, we also included controls for the levels of (i) regional digital infrastructures, (ii) regional digital skills and (iii) regional industrial specialization as well as the interactions between these regional controls and the pandemic severity measure. To compute the first two additional controls, we used Eurostat data. We measured regional digital infrastructures as the percentage of households in the region with broadband access (*Broadband*_*i*_) and regional digital skills as the percentage of residents in the region who ordered goods or services over the Internet for private use in the last 12 months (*DigitalSkills*_*i*_). Both variables were computed using 2019 data. To measure regional industrial specialization, we used the data extracted from the Infocamere Business Register and computed a Balassa index (Balassa, 1965) for each region-industry pair in 2019 (*BI*_*ij*_). The index was computed as the ratio between two shares. The numerator is the share of limited liability companies active in region *i* and industry *j* out of the total number of limited liability companies active in *i*. The denominator is the share of Italian limited liability companies active in *j* out of the total number of limited liability companies active in Italy. The values of the index are higher than 1 when region *i* is more specialized in industry *j* than the average Italian region, whereas the opposite holds true when the values of the index are lower than 1. The effects of our main explanatory variables on new firm creation are robust to the inclusion of *Broadband*_*i*_, *DigitalSkills*_*i*_, *BI*_*ij*_ and their interaction terms with the measure of pandemic severity.

Second, we excluded from the sample the less populated regions, i.e. those with population densities lower than 100 inhabitants per square metre, namely, Basilicata, Molise, Trentino, South Tyrol, Sardinia and Aosta Valley. The results of these estimates are in line with those discussed above. The only difference is the lower significance of the interacting term *ShutdownPolicies*_*ijt*_ × *COVID19Severity*_*it*_*.*

Third, as discussed in Sect. 5, the variable *COVID19Severity*_*it*_ is likely to underestimate the severity of the COVID-19 pandemic between March 2020 and May 2020. We, therefore, replicated our analyses by using alternative measures of pandemic severity. These are the average number of active COVID-19 cases (i.e. infected people whose most recent test was positive) in region *i* in month *t* per 1000 inhabitants in the region (*InfectedCases*_*it*_), the average number of infected people hospitalized in region *i* in month *t* per 1000 inhabitants in the region (*HospitalizedCases*_*it*_) and the average number of people in intensive care units in region *i* in month *t* per 1000 inhabitants in the region (*ICUCases*_*it*_). For all the alternative measures considered, the results of the estimates when focusing on the first wave of the pandemic are in line with the evidence reported in Table 6. It is fair to acknowledge that we also detected a negative and significant effect of pandemic severity in the second wave when using *InfectedCases*_*it*_.

## Discussion

In this study, we advance disaster research by empirically exploring the underinvestigated impact on new firm creation of an unprecedented disaster — the COVID-19 pandemic. Specifically, through econometric analyses using data on Italy in the first and second 2020 pandemic waves, we distinguish the effects of pandemic severity and pandemic-related policies.

Our contribution to extant literature is threefold. First, we advance the limited knowledge about the effects of disasters on entrepreneurial activity (for a recent work on the topic, see Edobor & Marshall, 2021). As we already mentioned, disaster research has not found a clear effect of disasters’ severity on new firm creation. The negative relationship we found between pandemic severity in a given region and new firm creation in that region in the first pandemic wave indicates that, in case of COVID-19, the severity of the disaster affected new firm creation at the local level.

Second, we provide a more nuanced understanding of the effects on entrepreneurial activity of disaster recovery policies (Chamlee-Wright & Storr, 2008). Our findings of the negative effects of both shutdown and firm support policies on new firm creation in the industries targeted by such policies are in line with disaster research claiming that disaster recovery policies hinder entrepreneurship. However, our finding of the positive effect of demand stimulus policies indicates that some recovery policies may be conducive to new firm creation as well.

The differences we found between the two pandemic waves as to the effects of pandemic severity and pandemic-related policies offer also a third contribution to disaster literature by providing further evidence of the effect of prior experience with disasters on behaviours. Indeed, while the negligible impact of pandemic-related policies during the second wave is probably attributable to the lower intensity of these policies as well as their lower degree of variation over time with respect to the first wave,^15^ the negligible effect of pandemic severity may be explained by the experience with the pandemic gained in the first wave. Until February 2020, nobody was familiar with COVID-19 in Italy; hence, the first pandemic wave caused emotional distress and anxiety in people, especially in the most affected regions. In turn, these feelings probably generated negative expectations of future profits by prospective entrepreneurs, making them less willing to create new firms. In the second wave, pandemic severity was comparable to severity in the first wave, but everyone already had experience with the pandemic. Hence, on the one hand, the emotional impact of the first wave might have (at least partially) worn off, and on the other hand, as the pervasive effects of the spread of the disease were probably clearer, the profit expectations of prospective entrepreneurs may have relied on the global pandemic situation rather than on the local situation. For these reasons, in the second wave, the pandemic severity in a given region likely had more negligible effects on the profit expectations of local prospective entrepreneurs and thus on new firm creation.

Disaster research has already shown that prior experience with disasters influences individuals’ behaviour in a disaster situation (Blanchard-Boehm & Cook, 2015). While less recent studies have shown that individuals with previous disaster experience are more likely to take mitigative actions (Burton & Kates, 1964; Olshansky & Kartez, 1998), more recent studies have shown that the effect of disaster experience on individuals’ behaviours is less straightforward. Prior experience is likely to affect individuals’ behaviours in two somewhat opposite directions: on the one hand, individuals with disaster experience tend to have a greater awareness of hazards, thus becoming more prone to take specific actions; on the other hand, individuals may become blasé about risks, thus reducing the probability of taking actions (Pan et al., 2020). These studies have focused on the experience developed by individuals hit by similar disasters in a short time span (e.g. recurring flooding or multiple hurricanes in a few years). Our results on the differences between the first and second pandemic waves suggest that even in prolonged disaster situations such as the COVID-19 pandemic, individuals develop an experience of the disaster, and this experience affects behaviours. However, we are unable to say whether the disaster experience that prospective entrepreneurs developed during the first wave of the COVID-19 pandemic made them more aware of the pandemic hazards, thus leading them to consider not only the local situation in making decisions on new firm creation in the second wave, or made them more accustomed to the disaster situation, thus reducing their likelihood to take into account differences in local severity of the pandemic when evaluating the option of creating new firms in a specific region.

## Conclusions

Despite this work contributes to advance disaster research, we are aware of its limitations, which open promising avenues for future research. First, in this study, we considered only some policy measures aimed at containing contagion and protecting the economy during the pandemic. Other policies were approved and implemented in Italy in 2020. For instance, to contain the contagion, in addition to imposing temporary shutdowns of nonessential economic activities, the Italian government imposed other types of restrictions on specific economic activities, such as limitations on the number of employees or customers simultaneously present in some facilities or reductions in service operating hours. Future works might thus extend our analysis by considering a wider set of pandemic-related policies. Second, in this work, we explored only the short-term effects of pandemic severity and pandemic-related policies on new firm creation. Analysing the evolution of new firm creation over a longer time horizon (e.g. until the first months after the end of the COVID-19 pandemic, whenever it will occur) will allow us to obtain a more comprehensive picture of the long-term effects of the pandemic and the associated disaster recovery policies. For instance, we might discover that although the loss in new firm registrations recorded in Italy in 2020 was not recovered in the short term, it may be fully recovered in the long term. Such a finding would provide evidence in favour of the argument already set forth by entrepreneurship research that in crisis times (nascent) entrepreneurs do not refrain from entering entrepreneurship; they merely delay new firm creation (Yu et al., 2009). Third, in this study, we explored the effects of pandemic severity and pandemic-related policies and their interactions, but we recognize that these effects may differ depending on the characteristics of the local economic system (e.g. local availability of financial resources and qualified human capital). Future works may thus investigate the moderating effect of local characteristics on the relationships explored in the current paper. Fourth, we focus our study on a specific country—Italy. Although this is an ideal context to investigate the effects on new firm creation of both the COVID-19 pandemic outbreak and the related policy actions (for a discussion on the context under scrutiny, see the Sect. 1), one may question whether the impact of the COVID-19 pandemic on new firm creation has been similar in other countries with different levels of pandemic severity and policy measures to halt the contagion. Finally, future works may examine other interesting topics related to new firm creation. For instance, scholars may explore inter-industry differences in firm creation and study to what extent the pandemic has stimulated new firm creation, thus contributing to the emergence of new industries. Alternatively, scholars may investigate whether profit expectations of prospective entrepreneurs (and, thus, new firm creation) in a specific region have been influenced not only by the pandemic severity in that region, but also by the pandemic severity in the neighbouring regions.

Despite the limitations discussed thus far, our findings have important policy implications. They suggest that policymakers can do a great deal to limit the negative effects of disasters on new firm creation. First, our results indicate that measures aimed at mitigating the impact of disasters, such as shutdowns imposed during the COVID-19 pandemic to contain the spread of the disease, inhibit new firm creation, but this negative effect can be alleviated by considering disaster severity when applying these measures. This is what the Italian government did during the second wave of the COVID-19 pandemic, by dividing regions into different categories based on how severe the pandemic was and implementing more severe restrictions in the regions with a higher contagion risk. If this approach had also been adopted during the first pandemic wave, it might have had less disruptive effects on new firm creation than the lockdown extended to the entire country. Second, our study may guide policymakers to better allocate resources to disaster recovery measures aimed at protecting the economy. In particular, in addition to providing relief to existing firms in the most severely hit industries, which are clearly important to avoid business failures, policymakers should simultaneously invest in stimulating demand in these industries, as such investments may be beneficial for both existing and new firms.

## Appendix

**Table 7:** List of policies designed by the Italian government to support firms or stimulate demand in specific industries

Decree laws	Firm support policies	Demand stimulus policies
Cura Italia Decree law (D.L. No 18/2020) (https://www.gazzettaufficiale.it/eli/id/2020/03/17/20G00034/sg)	•Support to cover interest expenses on loans for firms in agriculture and fisheries •Emergency fund for the entertainment, cinema and audiovisual industries •Grants for companies offering public bus services •Suspension of fee payment for sport sector •Tax credits for companies in the printing industry	•Grants to cover the costs of sanitation and disinfection of offices
Liquidity Decree law (D.L. No 23/2020) (https://www.gazzettaufficiale.it/eli/gu/2020/04/08/94/sg/pdf)		•Tax credits for the purchase of protective equipment in the workplace
Relaunch Decree law (D.L. No 34/2020) (https://www.gazzettaufficiale.it/eli/id/2020/05/19/20G00052/sg)	•Grants for small firms offering fuel distribution services on motorways •Investments in the tourism industry •Grants for newsstands and the publishing industry •Reliefs for the air, rail and naval transport sectors •Funding for the sport industry •Grants and reliefs for the agriculture, fisheries and aquaculture industries	•Grants for the purchase of cars •Tax credits for family holidays •Tax credits for the purchase of bicycles and vehicles for personal mobility with mainly electric propulsion •Tax credits for renovation/refurbishment works
August Decree law (D.L. No 104/2020) (https://www.gazzettaufficiale.it/eli/id/2020/08/14/20G00122/sg)	•Grants for restaurant and catering services •Grants for shops •Tax exemptions for tourism and entertainment sectors •Reliefs for cultural industries •Reliefs for the naval transport sector •Reliefs for the publishing industry	•Grants for the purchase of low-emission cars
Relief Decree laws (D.L. No 137/2020; 149/2020; 154/2020; 157/2020) (https://www.gazzettaufficiale.it/eli/id/2020/10/28/20G00166/sg) (https://www.gazzettaufficiale.it/eli/id/2020/11/09/20G00170/sg) (https://www.gazzettaufficiale.it/eli/id/2020/11/23/20G00175/sg) (https://www.gazzettaufficiale.it/eli/id/2020/11/30/20G00183/sg)	•Reliefs and tax exemptions for the sport sector •Reliefs and tax exemptions for tourism and cultural industries •Reliefs for the publishing industry •Reliefs for the organizers of conferences and fairs •Reliefs and tax exemptions for the agriculture, fisheries and aquaculture industries	•Grants for the purchase of anti-COVID-19 drugs

## Footnotes

1 It is worth acknowledging that pandemic severity can also stimulate new firm creation in few specific industries. First, pandemic severity may lead many existing firms to close. Such exits can stimulate new firm creation in some industries because new firms can take advantage of the resources freed by the exited firms, thus facing reduced market entry requirements (Pe’Er and Vertinsky, 2008; Monllor and Murphy, 2017). Second, in line with the argument that disasters may have also a “stimulus effect” by increasing demand for peculiar goods and services (Edobor and Marshall, 2021), pandemic severity may stimulate new firm creation in some industries because it either temporarily increases demand (as happened, e.g. in the food industry; Kemp et al., 2021) or provides opportunities to develop products/services able to ameliorate the disaster condition, which may find other uses once the pandemic will be over (as happened in telemedicine or online education, among others; Ruiz-Rosa et al., 2020).

2 To make this argument clearer, let us provide an example. In early December 2020, the Italian government ordered hotels located in mountain areas to shut down activities during Christmas holidays to avoid gatherings in mountain villages that may have contributed to the spread of the disease. As Christmas holidays are the peak period for mountain tourism, this shutdown policy had particularly negative effects on the expected revenues of hotels in these geographic areas. Hotel owners were sure they would be unable to recoup the revenues lost in the Christmas period in the following months.

3 The list of essential industries is available at the following link (in Italian): http://www.governo.it/sites/new.governo.it/files/documenti/documenti/Notizie-allegati/covid-19/dpcm_20200322_allegato_1.pdf (accessed on January 20, 2021).

4 All the policies implemented by the Italian government to contain the spread of the disease are described at these links (in Italian): http://www.governo.it/it/coronavirus-misure-del-governo and http://www.governo.it/node/14343 (accessed on January 20, 2021).

5 In addition to the restrictions imposed by the Italian government, Regional Authorities were allowed to adopt specific additional restrictive provisions of a local nature. These provisions are not considered in our analysis.

6 The economy recovery plans developed by the Italian government also include policy measures intended to support employees, families and citizens. In the present study, we do not consider these policies. Neither do we consider the policy measures developed by Regional Authorities because they are hardly comparable to the policies of the Italian government as the funds allocated to the former measures are much more modest than the funds allocated to the latter.

7 The only exception is a measure included in the August decree law (D.L. No 104/2020); money was allocated to support the start-up of new businesses by people under 30.

8 https://github.com/pcm-dpc/COVID-19 (accessed on March 25, 2021).

9 http://www.governo.it/it/coronavirus-normativa (accessed on January 20, 2021).

10 Results from the Hausman test (Hausman, 1978) for each regression presented in this study confirm the appropriateness of the fixed effects model against the random effect model.

11 During the second wave of the pandemic, different shutdown policies were implemented in Trentino and in South Tyrol, despite these two areas belonging to the same administrative region. Therefore, in the econometric analysis, we consider these two areas as separate regions.

12 The idea that *COVID19Severity*_*it*_ underestimates the overall effect of the pandemic is confirmed by data on the overall number of deaths occurred in these months. Indeed, data from the Italian National Institute of Statistics (https://www.istat.it/it/files//2020/03/Nota-informativa-dati-mortalita-al-30-settembre.pdf) indicate that the number of deaths from any causes between March 2020 and May 2020 was much higher than the mortality observed between March and May in the previous years. The excess observed in overall mortality in the March 2020–May 2020 period noticeably exceeded the number of deaths registered by the Civil Protection Department as COVID-19-related.

13 The main differences between the two waves of the pandemic are in regard to the geographical distribution of the most severely hit areas, the number of industries targeted by shutdowns and the length of shutdowns and the time distribution of firm support and demand stimulus policies. The differences between the two waves are discussed in the concluding section of the paper.

14 For the sake of brevity, here, we do not report the results of the robustness checks. These are available from the authors upon request.

15 In the second wave, shutdown policies were focused on fewer industries than in the first wave, while firm support and demand stimulus policies allocated significantly less funding. Moreover, we observed fewer changes in pandemic-related policies than in the first wave. Indeed, in many industries (e.g. the entertainment industry, the sports industry), shutdown policies were in force starting from October until the end of the wave, while changes in firm support and demand stimulus policies occurred only in October (whereas in the first wave, such policies were approved in multiple months).
